# Multivariate sib-pair linkage analysis of longitudinal phenotypes by three step-wise analysis approaches

**DOI:** 10.1186/1471-2156-4-S1-S68

**Published:** 2003-12-31

**Authors:** Zheng Guo, Xia Li, Shaoqi Rao, Kathy L Moser, Tianwen Zhang, Binsheng Gong, Gongqing Shen, Lin Li, Ruth Cannata, Erich Zirzow, Eric J Topol, Qing Wang

**Affiliations:** 1 Department of Computer Science, Harbin Institute of Technology, Harbin, China; 2 Department of Biomedical Engineering, Biomathematics and Bioinformatics, Harbin Medical University, Harbin, China; 3 Center for Cardiovascular Genetics, Department of Cardiovascular Medicine, the Cleveland Clinic Foundation, 9500 Euclid Avenue, Cleveland, Ohio, USA; 4 Department of Molecular Cardiology, Lerner Research Institute, The Cleveland Clinic Foundation, 9500 Euclid Avenue, Cleveland, Ohio, USA; 5 Department of Medicine, Institute of Human Genetics, University of Minnesota, Minnesota, USA

## Abstract

**Background:**

Current statistical methods for sib-pair linkage analysis of complex diseases include linear models, generalized linear models, and novel data mining techniques. The purpose of this study was to further investigate the utility and properties of a novel pattern recognition technique (step-wise discriminant analysis) using the chromosome 10 linkage data from the Framingham Heart Study and by comparing it with step-wise logistic regression and linear regression.

**Results:**

The three step-wise approaches were compared in terms of statistical significance and gene localization. Step-wise discriminant linkage analysis approach performed best; next was step-wise logistic regression; and step-wise linear regression was the least efficient because it ignored the categorical nature of disease phenotypes. Nevertheless, all three methods successfully identified the previously reported chromosomal region linked to human hypertension, marker GATA64A09. We also explored the possibility of using the discriminant analysis to detect gene × gene and gene × environment interactions. There was evidence to suggest the existence of gene × environment interactions between markers GATA64A09 or GATA115E01 and hypertension treatment and gene × gene interactions between markers GATA64A09 and GATA115E01. Finally, we answered the theoretical question "Is a trichotomous phenotype more efficient than a binary?" Unlike logistic regression, discriminant sib-pair linkage analysis might have more power to detect linkage to a binary phenotype than a trichotomous one.

**Conclusion:**

We confirmed our previous speculation that step-wise discriminant analysis is useful for genetic mapping of complex diseases. This analysis also supported the possibility of the pattern recognition technique for investigating gene × gene or gene × environment interactions.

## Background

The search for efficient and powerful statistical methods and optimal mapping strategies for complex human diseases that are categorical in nature continues to be one of the main tasks faced by genetic epidemiologists. Sib-pair linkage analysis is one of the most popular methods (designs). The possible statistical methods for sib-pair linkage analysis of categorical human diseases include linear regression (e.g., Haseman-Elston regression), generalized linear models (e.g., logistic regression), and the novel pattern recognition techniques (e.g., neural network [1] and discriminant analysis [2,3]). Haseman-Elston linear regression was originally proposed to analyze continuously distributed traits. Nevertheless, application of linear regression to discrete traits has been successful [4] due to its robustness to the departure from normality and large sample theory. The discriminant analysis proposed by us recently is in essence anti-traditional in that the positions of the components in the genetic model are reversed, i.e., we believe that the variation in marker identity by descent (IBD) among the sib-pairs is due to the classification of the phenotypes of a sib pair, for example, concordant affected, discordant, and concordant unaffected. This novel multivariate approach has several unique characteristics in the context of linkage analysis. First, the group variable for classification of affection status of a sib pair is no longer the 'response' variable as in the conventional modelling, but is the explanatory variable instead for the differential multivariate distributions over the feature space. Second, no distribution assumption for the grouping variable is assumed because it is considered to be fixed (constant) in the discriminant analysis, while a multivariate normal distribution is often imposed on the feature variables within a group. Third, it can have very distinct statistical properties from the conventional (generalized) linear models, for example, there is not a balanced design for sib-pair linkage analysis and the statistical power for linkage analysis of a binary disease can be higher than the corresponding ordinal traits [2,3]. In the previous papers [2,3], we had studied some properties of the discriminant sib-pair linkage analysis approach via simulation and an application to a simulated disease for Genetic Analysis Workshop 12 (GAW12). In this study, we further investigated its properties and performance by applying it to the chromosome 10 data from the Framingham Heart Study and by comparing it with step-wise logistic regression and linear regression.

## Methods

### Data preparation

We used the summary method proposed by Levy et al. [5]. Two hypertension phenotypes, systolic blood pressure (SBP) and high blood pressure (HBP), were examined in this study. First, means of the original longitudinal measures (up to 21 and 5 repeated measures for the original and the offspring cohort, respectively) for the two phenotypes were obtained for each cohort. We called them continuous SBP and HBP, respectively. Then, these continuous phenotypes were truncated into categories. The binary SBP for each subject was coded as affected if the mean SBP ≥ 140 and unaffected otherwise. The binary HBP for each subject was coded as affected if mean HBP ≥ 0.5 (equivalent to half of the examinations that were diagnosed as hypertension) and unaffected otherwise. The trichotomous HBP was obtained by applying two cut points (0.33 and 0.67). The three categories correspond to  and  times of examinations (up to 21 and 5 for the original and the offspring cohort, respectively) diagnosed to be hypertensive. This type of phenotypic partition is analogous to a clinical scoring system [6] to assess the evidence supporting fulfillment of a given hypertension criterion because multiple diagnoses might provide more definitive information on manifestations possibly consistent with the inherent pathological state of a patient. The three categorical scores can be interpreted as the degree of confidence for classification of a patient based on multiple diagnoses. Mean summaries for nine epidemiological risk factors for hypertension were also obtained and modeled simultaneously with linkage (marker IBD) or not modeled. The nine covariates were the longitudinal means of total cholesterol (CHOL), cigarettes per day (CPD), alcohol (grams/day) (DRINK), fasting glucose (GLUC), high density lipoprotein (HDL), height (HGT), hypertensive treatment (HRX), triglycerides (TRIG), and weight (WGT). To be consistent among the three step-wise approaches, all the conditions (phenotypic data, markers, covariates, and variable selection criteria (*P *= 0.05)) were kept identical.

### Step-wise discriminant analysis (STEPDISC)

The methodological details were described previously [2,3]. The feature variables include the estimated proportions of alleles shared IBD by the sib pair at each marker on chromosome 10, obtained from S.A.G.E. GENIBD [7], and the nine covariates. For a binary trait, the three groups were defined as concordantly unaffected sibs, discordant sibs, and concordantly affected sibs. The groups for the trichotomous HBP were defined similarly, resulting in six mutually exclusive groups and each representing a specific combination of two ordinal values of a sib-pair. To assess the contribution from each feature variable, we used the SAS step-wise discriminant analysis procedure [8] via an F statistic. The statistical significance for each feature variable was determined by its partial contribution to the partition of the observed affection groups, with the presence of other features in the finally selected subset.

### Step-wise logistic regression (STEPLOG)

The group variable was considered as an ordinal (or binary) dependent variable and the feature vector (multiple marker IBD estimates and covariates) as the independent variables. A nonlinear relationship (logit) between the dependent and independent variables was taken. For binary data (collapsing concordantly affected or unaffected sib pair into one group), a conventional logistic regression was used. For ordinal data, the SAS LOGISTIC [8] procedure fits a parallel lines regression model based on the cumulative distribution probabilities of response categories, rather than on their individual probabilities. The statistical *P*-value for each (selected) independent variable was obtained from the final model fitting with only selected variables included via an asymptotic chi-squared statistic.

### Step-wise linear regression (STEPREG)

We extended the new version of Haseman-Elston regression by including multiple marker information and the analysis proceeds in a step-wise manner. The binary disease phenotypes were taken as if they were continuous, i.e., by giving "affected" and "unaffected" different quantitative scores-without loss of generality, 1 and 0, respectively. Then, the centered cross-product of two sibs' phenotypic values was linearly regressed onto the proportion of alleles that the sibs shared IBD at markers. The covariates were coded similarly to that for the dependent variable and fitted in the regression for adjustment. The SAS REG [8] was used to perform a step-wise linear regression and statistical *P*-values for the finally included predictors, determined by an F statistic, were reported.

## Results

### Genetic linkage evaluation by three different statistical methods

The summary of linkage analysis of longitudinal SBP and HBP, with and without adjusting the hypertension risk factors as well as assessment of these risk factors using three different approaches, is given in Tables 1,2,3. Only those sib pairs (about 500) with data for all covariates and marker IBDs could be used. For most traits, all three methods identified the significant linked region, marker GATA64A09, which is consistent with the results reported in [5]. In terms of statistical significance of detection of linkage, STEPDISC was better than other two methods. Because STEPREG ignored the discrete nature of disease phenotypes, it was inferior to STEPLOG, as we expected. Generally, taking into account the hypertension risk factors reduced the statistical efficiency (in terms of statistical significance) for all the methods, suggesting the existence of interactions between these environment factors and the genetic linkage components. Consistent results for assessing risk factors with STEPDISC and STEPLOG were observed. The significant effect of antihypertensive treatment on blood pressure and hypertension was identical among the three methods.

### Exploration of gene × gene and gene × environment interactions

Due to the sequential nature of the partial F statistics used in STEPDISC, we can easily infer the joint actions of two effects (interactions), as demonstrated in the following. Table 4 lists the dynamic changes of F statistics in the step-wise selections (Step 1 to Step 5). Comparison of the column Step 2 (the conditional contribution of the rest of features on the effects of HRX being taken into account) with Step 1 (the marginal contribution of each feature on separating disease affection groups of sib pairs) indicates that CHOL, CPD, DRINK, GLUC, HDL, HGT, but not TRIG and WGT, have interactions with HRX. Scrutiny of dramatic changes for F statistics for markers after removing the effects of HRX revealed the existence of possible gene × environment interactions under the assumption of existence of a gene(s) for hypertension on this chromosome. The fact that accounting for the effects of marker GATA64A09 leads to a significant drop in F statistic for GATA115E01 (3.56 to 1.48, that is, the ability of partitioning the sib pairs into 'correct' affection groups based on marker GATA115E01 IBDs, is greatly reduced by simultaneously adjusting for the effects of marker GATA64A09) suggests a gene × gene (marker × marker) interaction between the two regions, which may be due to close linkage or epistasis.

### Is a trichotomous phenotype more efficient than a binary one?

**Figure 1:**
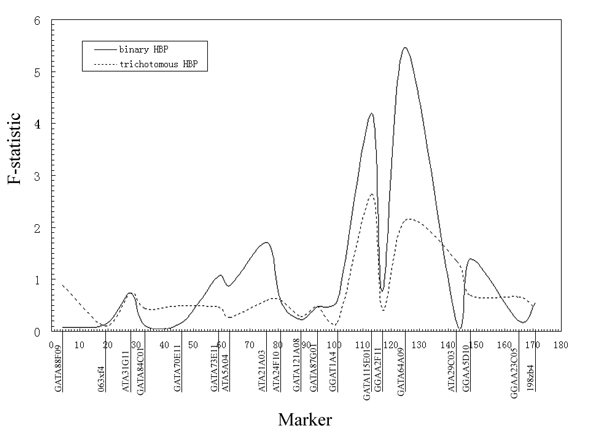
Chromosome 10: F-statistic profiles for binary high blood pressure (HBP) and trichotomous HBP, respectively

It is a generally accepted notion that an ordinal (with more than two categories) phenotype contains more information than binary data and thereafter is more efficient for linkage analysis if a generalized linear model is used [4]. However, the notion may not be true for discriminant linkage analysis [3]. To further validate our previous finding [3], we compared the linkage statistic profiles for binary and trichotomous HBPs (Figure 1), obtained using step-wise discriminant analysis. It is evident that for most of the markers, the statistic for trichotomous HBP is much lower than that for the corresponding binary trait. Subsequent discriminant linkage analyses supported our speculation: when trichotomous HBP was used, the marker GATA64A09 was no longer detectable; on the contrary, STEPLOG (a generalized-linear-model-based approach) did identify this region (*P *= 0.0414).

## Discussion

Although most findings for the utility, performance and properties of STEPDISC in this study were also confirmed through a large simulation, and an application to a simulated disease for GAW12 [2,3], we view this analysis as exploratory because of the simplistic approach presented here and encourage further studies on the following issues. We did not address the issue of correlated IBDs in the large sibships, which violates the assumption of independence for STEPDISC as well as for other two methods. In addition, the reported SAS *P*-values might be liberal and might deviate from the true chromosome-wide *P*-values, as suggested by our simulations [3]. Furthermore, STEPDISC cannot distinguish well between close linkage and epistasis for evaluation of gene × gene interactions.

We have compared three typical sequential statistical methods for genetic mapping of complex diseases. Genetic analyses for categorical traits are known to be difficult because phenotype cannot be described by a linear function of genetic and environmental effects. In the sib-pair-based linkage analysis, the very act of taking a quadratic form of sibs' phenotypic values has changed the relationship between the model components and renders the relationship unclear. Several issues for sib-pair based linkage analysis deserve our attention. First, what kind of relationship, linear or nonlinear, should be taken to describe the relationship between the new phenotype and its determinant, IBD values? Fortunately, our proposed discriminant sib-pair-linkage analysis does not require explicitly specifying this relationship and thus tactically avoids this difficulty. Second, how do we rank affection groups? Is the order important? To answer this question, we phenotyped the binary diseases (binary SBP and HBP) by giving "affected" a value of 0 and "unaffected" a value of 1 so that for logistic regression based linkage analysis, concordantly affected sib pair was coded as 0 and concordantly unaffected sib pair was coded as 2. Identical results were obtained for all the three methods (data not shown), indicating that interchanging the positions for two concordant groups has no effect for sib-pair linkage analysis of binary diseases. It can be easily shown that the new coding for marginal phenotypes does not change the numerical values of the centralized cross-product for the three affection groups, but a rigid mathematical proof is needed for logistic modelling. Finally, we conducted additional analysis to investigate the effects of collapsing two concordant groups into a single group. Using the collapsed two-group data, we did not identify a single marker to be significantly linked to hypertension phenotypes using all the three methods (data not shown), suggesting that this common collapsing practice lead to loss of statistical efficiency.

## Conclusions

Step-wise linear regression, logistic regression, and discriminant analysis are three representatives of sequential statistical methods that are potentially useful for sib-pair linkage analysis of complex human diseases. All the methods successfully identified the previously reported linked region, marker GATA64A09, at the chromosome-wide significance level of 0.05. However, from both theoretical and applied views, step-wise discriminant analysis appears to be the most efficient for sib-pair linkage studies. This conclusion was supported by this and the previous studies [2,3]. Further investigations on the possibility of using this data mining technique for detecting gene × gene and gene × environment interactions under sib-pair designs are encouraged.

## Figures and Tables

**Table 1:** Summary^A ^of linkage analysis of longitudinal SBP and HBP using three statistical methods.

Trait	Method					
	
	STEPDISC		STEPLOG		STEPREG	
			
	Marker	*P*	Marker	*P*	Marker	*P*
Continuous HBP					GATA64A09	0.0282
Binary HBP			GGAA2F11	0.0444		
	GATA64A09	0.0044	GATA64A09	0.0067		
					198ZB4	0.0195
Continuous SBP					GATA64A09	0.0028
Binary SBP	GATA64A09	0.0029	GATA64A09	0.0096	GATA64A09	0.0485

^A ^Linkage was analyzed without the presence of the hypertension risk factors.

**Table 2:** Summary^A ^of linkage analysis of longitudinal SBP and HBP using three statistical methods.

Trait	Method					
	
	STEPDISC		STEPLOG		STEPREG	
	
	Marker	*P*	Marker	*P*	Marker	*P*
Continuous HBP					GATA115E01	0.0463
Binary HBP					GATA115E01	0.0255
	GATA64A09	0.0118	NULL	-	GATA64A09	0.0289
Continuous SBP					GATA64A09	0.0167
					GGAA5D10	0.0235
Binary SBP	GATA64A09	0.0086	GATA64A09	0.0149	GATA64A09	0.0101

^A^Linkage was analyzed in the presence of the nine hypertension risk factors (the longitudinal means of total cholesterol, cigarettes per day, alcohol (grams/day), fasting glucose, high density lipoprotein, height, hypertensive treatment, triglycerides and weight).

**Table 3:** Assessment of hypertension risk factors

	Method					
	
Trait	STEPDISC		STEPLOG		STEPREG	
	
	Feature	*P*	Factor	*P*	Factor	*P*
	
Continuous HBP					HRX	<0.0001
					CHOL	0.0335
					GLUC	0.0316
					HGT	0.0155
Binary HBP	HRX	<0.0001	HRX	<0.0001	HRX	<0.0001
	CHOL	0.0001	CHOL	0.0073		
	GLUC	0.0070	GLUC	0.0203	GLUC	0.0011
					HGT	0.0092
Continuous SBP					HRX	<0.0001
					WGT	0.0003
Binary SBP	HRX	<0.0001	HRX	<0.0001	HRX	<0.0001
	CHOL	<0.0001	CHOL	<0.0001		
					WGT	0.0297

^A^The nine hypertension risk factors evaluated are the longitudinal means of total cholesterol (CHOL), cigarettes per day (CPD), alcohol, grams/day (DRINK), fasting glucose (GLUC), high density lipoprotein (HDL), height (HGT), hypertensive treatment (HRX), triglycerides (TRIG), and weight (WGT).

**Table 4:** Evaluation of gene×gene, gene×environment and environment×environment interactions for binary HBP, using step-wise discriminant analysis.^A^

	F Statistic				
	
Feature	Step 1	Step 2	Step 3	Step 4	Step 5
CHOL	13.92	10.44	inclusion	inclusion	inclusion
CPD	1.00	0.18	0.44	0.44	0.50
DRINK	3.54	3.24	1.97	1.96	2.06
GLUC	12.86	6.16	4.85	inclusion	inclusion
HDL	1.32	0.17	0.08	0.06	0.05
HGT	5.64	2.07	2.1	3.18	2.98
HRX	112.68	inclusion	inclusion	inclusion	inclusion
TRIG	0.00	0.07	0.96	1.99	1.88
WGT	3.67	3.72	2.78	2.26	2.21
GATA115E01	4.2	3.47	3.1	3.56	1.48
GGAA2F11	0.78	0.81	0.62	0.68	0.85
GATA64A09	5.46	4.32	4.32	4.48	inclusion
ATA29C03	0.17	0.07	0.06	0.02	0.37

^A^Some markers that are essentially not affected by interactions are deleted from the table.
